# Treatment Strategies and Future Directions for Locally Advanced Pancreatic Cancer

**DOI:** 10.3390/cancers18213520

**Published:** 2026-11-02

**Authors:** Kevin M. Turner, Jason B. Gurewitz, Jessica M. Frakes, Jason W. Denbo, Mokenge P. Malafa, Pamela J. Hodul, Dae Won Kim, Andrew J. Sinnamon

**Affiliations:** 1 Department of Gastrointestinal Surgical Oncology and Gastroenterology, H. Lee Moffitt Cancer Center & Research Institute, 12902 USF Magnolia Drive, Tampa, FL 33612, USA; 2 Department of Radiation Oncology, H. Lee Moffitt Cancer Center & Research Institute, Tampa, FL 33612, USA; 3 Department of Gastrointestinal Medical Oncology, H. Lee Moffitt Cancer Center & Research Institute, Tampa, FL 33612, USA

**Keywords:** locally advanced pancreatic cancer, systemic therapy, locoregional therapy, future directions

## Abstract

Locally advanced pancreatic cancer is a complex clinical entity, combining a locally aggressive tumor in a disease marked by systemic failure. Accordingly, systemic therapy upfront is recommended, with translation of recent advances in the metastatic setting promising to revolutionize the care of locally advanced disease in the future. In those patients where systemic disease remains controlled, locoregional therapies including surgery and radiation may play a role. Specific considerations of these therapies are presented in a disease phenotype marked by invasion into surrounding vasculature/luminal organs. Ongoing investigations are set to define the role of novel therapies in locally advanced disease, some of which have already gained approval. With recent advances in the control of systemic disease, locally advanced disease represents a prime target for study and treatment optimization to improve outcomes for patients.

## Simple Summary

The management of locally advanced pancreatic cancer (LAPC) requires multi-disciplinary involvement, with systemic therapy as the backbone of current treatment paradigms. In this review we discuss the initial workup of LAPC and appropriate restaging. Considerations for optimal systemic therapy regimens are reviewed with special emphasis on future directions with targeted therapy. The clinical data supporting the role of locoregional therapies, including surgery and radiation, as well as potential novel treatments are discussed. Finally, future directions and advances in the management of LAPC are outlined.

## 1. Introduction

Pancreatic cancer is estimated to impact 67,530 patients in 2026, representing 3.2% of all cancers in the US; however, it is predicted to account for 8.4% of all mortalities [1]. Pancreatic ductal adenocarcinoma (PDAC) is the most common type of pancreatic cancer, accounting for more than 90% of cases. PDAC develops primarily through two precursor pathways: pancreatic intraepithelial neoplasia and intraductal papillary mucinous neoplasms. Symptoms are often delayed and presentation most commonly occurs in advanced stages where outcomes remain poor (overall 5 yr OS: 13.7%) [1]. While the majority of patients are unable to undergo surgery due to extent of disease at diagnosis, the best long-term outcomes have been consistently shown in patients who undergo surgical resection as part of multimodal therapy [2,3]. The focus of this narrative review is locally advanced pancreatic cancer (LAPC), classified as a localized PDAC that is initially unresectable by nature of vascular involvement, necessitating a multimodal approach to treatment. LAPC deserves its own discussion due to complex locoregional management implications in a disease marked by systemic failure.

## 2. Staging and Response Assessment

The majority of patients with PDAC, ~50–57%, present with metastatic disease where surgical resection is not indicated [4,5]. Another 10–23% of patients present with resectable disease, where both upfront surgery and perioperative therapy are supported by guidelines [6]. The decision to pursue perioperative therapy in resectable disease is based on the biologic risk of systemic recurrence. The remaining 18–35% have locally advanced or borderline resectable pancreatic cancer (BRPC) where multimodal therapy is always recommended. BRPC and LAPC are distinguished based on the degree and type of tumor–vessel interface involved, with different classification criteria from different guidelines/societies. However, in general, BRPC is classified as reconstructible vein involvement or arterial involvement < 180° while LAPC is classified as unreconstructible venous involvement or arterial involvement > 180°, with specific anatomic exceptions. (Table 1) In a large Dutch series of more than 16,000 patients, LAPC accounted for 10.9% of cases, which would equate to ~7000 new cases in the US every year, demonstrating the scale of this clinical problem [5]. LAPC has been proposed to be further classified into LAPC A and B, which further stratifies by increasing arterial involvement (LAPC A: 180–270° and LAPC B: >270°) and is associated with the ability to complete multimodal therapy [7]. These definitions are predicated on the understanding that increasing vessel involvement is associated with a decreased ability to achieve margin negative resection; of paramount importance to the efficacy of surgery in PDAC, and a potentially increased risk of occult micro-metastatic disease [8,9,10].

Pancreatic cancer failure is systemic in the vast majority of patients, with pre-clinical evidence suggesting metastatic disease is present at diagnosis in most patients [11,12,13]. Therefore, defining metastatic risk and ability to tolerate therapy is as important as tumor anatomy, collectively known as the *ABC* factors of localized pancreatic cancer (*A*natomy, *B*iology, *C*ondition/*C*omorbidities) [14,15]. CA 19-9 is the most widely used biomarker in PDAC, with normalization seen as an important oncologic prognostic factor in both patients with upfront surgery and in those who undergo neoadjuvant treatment [16,17]. However, 5–10% of patients are non-secreters due to a Lewis-negative phenotype. Duke pancreatic monoclonal antigen type 2 (DUPAN-2) has recently been identified as a surrogate biomarker in these patients with normalization after neoadjuvant therapy and post-resection also associated with improved outcomes [18]. Additional signs/symptoms associated with advanced disease include: excessive weight loss, uncontrolled pain, large primary tumors, equivocal/indeterminate imaging characteristics, and regional lymphadenopathy [4,6]. As important as anatomy and biology is the underlying performance status of the patient and any pre-existing comorbidities which have been shown to be associated with survival [19].

Accurate staging is critical to selection, with guidelines stressing high-quality cross-sectional imaging including CT pancreatic protocol and CT chest, biochemical evaluation, and genetic testing (germline-recommended; somatic-preferred) [6,20]. Diagnostic laparoscopy is recommended for patients with LAPC by the Operative Cancer Standards and the NCCN guidelines, with radiographically occult disease found in 10–18% of patients (~14% gross metastatic disease, ~4% cytology positive) [21,22]. Proposed risk factors for positive diagnostic laparoscopy include large size, body/tail lesions, high CA 19-9, extrapancreatic extension, and young age [21]. PET scan is considered by the NCCN guidelines in those with high-risk features for metastatic disease [6]. However, PET has also been advocated to quantify treatment response among patients undergoing extensive surgical resections [17,23,24]. Recently published guidelines by REDISCOVER consensus conference for the perioperative care of patients with locally advanced disease provides an excellent overview to perioperative care [25].

Pre-operative therapy in LAPC may be considered a misnomer, as the majority of patients do not undergo surgery [26]. Rather, it should be thought of as upfront systemic therapy, with a goal of potentially undergoing conversion surgery, defined as “surgical resection with curative intent after non-surgical induction therapy following a favorable response in patients with upfront unresectable disease” [13,24]. Favorable response is suggested by significant reduction/normalization of CA 19-9, radiographic disease demonstrating at least stable disease if not improvement in tumor–vessel interface, improvement in symptomatology (i.e., pain, weight loss), and maintenance of adequate performance status for surgical resection. In prospective clinical trials, resection rates in LAPC have been reported between 4–36% with R0 rates of 25–67% [27,28,29,30]. Increasing arterial involvement is critical, as resection rates in LAPC A are reported to be as high as 62%, compared to only 24% in LAPC B [7]. Of those that progress to surgical exploration with LAPC, only 62% undergo potentially curative resection (removal of the tumor with goal of margin negative resection, R0 resection) [31]. The most common reason for aborted resection in LAPC is local unresectability, which differs from resectable and BRPC where occult metastatic disease is the more common cause. Further, RECIST responses to upfront systemic therapy are rare (~28% downstaging) and imaging responses often underappreciate resectability due to difficulty in distinguishing viable tumor from treatment-related fibrosis [17,32]. Chemotherapeutic switch is recommended in cases where progression is noted that does not preclude surgery [33]. Multi-disciplinary evaluation to determine resectability is critical, as inter-observer discrepancy on local staging is common [34]. Following completion of pre-operative therapy, repeat staging with consideration for repeat diagnostic laparoscopy should be considered.

## 3. Systemic Considerations

Systemic therapy is recommended for all patients with PDAC, optimally up to a duration of 6 months; however, systemic therapy in LAPC requires personalization of therapy in line with disease response [6,35]. Historically, this was performed in the adjuvant setting; however, due to difficulties with receipt of multi-agent chemotherapy postoperatively (~50% or less), early treatment of micro-metastatic disease, and potential sterilization of the surgical margin/downstaging, upfront systemic therapy is conditionally recommended for resectable disease and strongly preferred for BRPC and LAPC [6,20,34,36]. Best practices for the delivery of upfront systemic therapy have been advocated to include delivery of therapy, frequent restaging, management of complications, and implications for surgery and pathology [37].

The NCCN preferred regimens in patients with LAPC are: gemcitabine/albumin-bound paclitaxel (GA), modified leucovorin/fluorouracil/irinotecan/oxaliplatin (mFOLFIRINOX/FOLFIRINOX), and liposomal irinotecan/fluorouracil/leucovorin/oxaliplatin (NALIRIFOX) [6]. These recommendations come from extrapolations from the metastatic setting [38,39,40]. Additional data specific to LAPC exist with a small number of prospective trials described below.

The NEOPAN trial was a phase III trial including patients with only LAPC which showed improved progression-free survival (mPFS: 9.7 mo vs. 7.7 mo, HR: 0.7, 95%CI: 0.5–1.0, *p* = 0.04), but not median overall survival (mOS: 15.7 mo vs. 15.4 mo), among patients treated with FOLFIRINOX compared to single-agent gemcitabine [41]. The NEOPAN trial demonstrated the superiority of FOLFIRINOX in LAPC, meeting its primary endpoint of improved PFS. However, the lack of OS benefit and increased toxicity associated with FOLFIRINOX suggest that gemcitabine remains a reasonable option for patients with LAPC and impaired performance status. GA has been evaluated in LAPC with time to treatment failure as the primary endpoint [28]. In this study, the median time to treatment failure, PFS and OS were 9 months (90% CI: 7.3–10.1), 10.9 months (90% CI: 9.3–11.6) and 18.8 months (90% CI: 15.0–24.0), respectively. After induction GA therapy, 20 patients (19%) became eligible for surgery, and 17 patients (16%) ultimately underwent surgery with 7 patients who achieved R0 resection [28]. In the NEOLAP trial, a phase II study, GA was compared to GA then FOLFIRINOX with equivalent conversion to surgical resection between arms (35.9% vs. 43.9%) [29]. Greater histopathologic downstaging was seen in the GA then FOLFIRINOX arm as a secondary outcome. Another key finding from this trial was the critical importance of R0 resection. The best survival was seen with R0 resection, and no difference was seen between margin positive resection and chemotherapy alone, underscoring the importance of proceeding with surgical resection when margin-negative resection is felt to be feasible. Finally, LAP07 and CONKO-007 are two phase III studies combining induction chemotherapy and chemoradiation compared to continuation of systemic therapy [27,30]. These data demonstrated equipoise in terms of overall survival between these strategies (discussed in radiation section). However, one systemic therapy consideration from the LAP07 trial was the lack of an efficacy signal in the gemcitabine/erlotinib arm [30].

Beyond cytotoxic chemotherapy, targeted therapies currently play a role in metastatic pancreatic cancer care, and logically these may be applied to LAPC in the future. In pancreatic cancer, somatic mutations in *KRAS* dominate the genetic landscape (~88%), followed by mutation in *TP53* (61–74%), *CDK2NA* (16–44%), and *SMAD4* (20–22%) [42]. *KRAS* wild-type tumors account for the remaining patients, with 20–30% of these patients harboring actionable fusions in *ALK*, *BRAF*, *MET*, *FGFR2*, *NRG1*, *RET*, *ROS1*, and *NTRK* [43,44,45]. Genetic syndromes account for only 10–15% of all pancreatic cancer, but harbor potentially actionable alterations such as platinum-based chemotherapy and maintenance *PARP* inhibitors with homologous recombination deficiency (ie. *BRCA 1/2*, *PALB2*) and immunotherapy in deficient mismatch repair tumors (i.e., *MLH1*, *MSH2/6*, *PMS2*). Proof of concept with precision medicine in pancreatic cancer was provided by the Know Your Tumor Registry Trial, which demonstrated increased PFS when matched therapy was provided [46]. While this approach is currently restricted to the metastatic setting, the recent paradigm shift with publication of the RASolute 302 trial may signal imminent integration into all phases of care due to its impressive efficacy and safety data [47]; treatment with a tri-complex RAS (ON) inhibitor, daraxonrasib, demonstrated doubling of progression-free and overall survival and tripling of objective response rates (ORR). If applied in the LAPC setting, these results could plausibly increase the pool of patients who become amenable to surgery [13]. Novel clinical trial designs will be required to integrate these new targeted therapies to determine optimal combination and sequencing [26,48].

## 4. Locoregional Management

### 4.1. Surgery

Generally, patients with LAPC are considered not surgically resectable at diagnosis due to extensive involvement with venous and/or arterial structures that preclude the ability to achieve a margin-negative resection. However, a minority of patients with excellent response to systemic therapy may convert to potentially resectable. Therefore, restaging cross-sectional imaging with high resolution and multiphase imaging is critical in order to identify those who may be amenable to surgical resection after initial systemic therapy (e.g., pancreatic protocol CT). For cases that become potentially resectable with surgery, vascular resection and reconstruction is commonly needed in order to achieve R0 resection. In these cases, accurate cross-sectional assessment of peripancreatic vascular anatomy is critical to operative success. High-quality imaging should provide the location, and relationship to named vessels, with a special emphasis on the first mesenteric arteries/veins and the pancreaticoduodenal arcades (Table 2). Special consideration should be paid to the following:

- Presence of celiac stenosis/median arcuate ligament syndrome when the gastroduodenal artery is sacrificed [49].
- Inferior pancreaticoduodenal artery caliber prior to planned distal pancreatectomy with celiac axis resection [50].
- Concept of a suitable target proximally and distally devoid of tumor involvement and amenable to anastomosis when vascular resection is planned (i.e., reconstructibility of the involved vascular system) [51].
- Cavernous transformation and the need for pre-operative radiologic decompression/intra-operative meso-caval shunt [51].

These are just some examples unique to LAPC clinical scenarios requiring pre-operative assessment to help formulate an operative plan.

**Table 2:** Pre-operative Vascular Considerations for Resection of Locally Advanced Disease.

Arterial Considerations	Venous Considerations
Celiac involvement	SMV/PV Involvement - Occlusion and collateral formation - Length of Involvement - Extension to jejunal/ileal branches
Common Hepatic Involvement - Extension onto proper hepatic	Left-Sided Drainage - Inferior mesenteric vein drainage - Coronary drainage
Superior Mesenteric Involvement	First Jejunal Vein - Relationship to SMA - Inferior pancreaticoduodenal vein insertion
Aberrant Arterial Involvement - Michel Classification Type	Gastrocolic Trunk Composition - Superior right colic vein - Right gastroepiploic vein - Anterior superior pancreaticoduodenal vein - Middle colic vein
Additional Arteries of Note - Inferior pancreaticoduodenal artery - First jejunal artery - Dorsal pancreatic artery	

Recently, a push towards total mesopancreas excisions and triangle clearance as techniques to completely extirpate tissue at-risk for recurrence has been seen in the literature. The mesopancreas is now defined as a retropancreatic compartment of lymphatic, neurovascular, and adipose tissue that is a common site of residual disease and bounded by specific structures, with excision of this area often called a total mesopancreas excision [52]. The triangle operation similarly involves removal of all neurolymphatic tissue between the celiac artery, superior mesenteric artery (SMA), and mesenteric–portal axis [53]. These operations have been shown to be effective in retrospective series; however, prospective data are lacking [54]. Artery-first approaches allow for early determination of resectability prior to an irreversible step, early division of the IPDA to decrease intra-operative blood loss, and clearance of the SMA margin [55]. There are at least six contemporary SMA-first approaches that have been described, with unique benefits and pitfalls to each approach. In the MAPLE-PD prospective trial comparing one SMA-first approach (mesenteric approach = infracolic SMA approach) to conventional surgery, both arms performed similarly with respect to survival (39.3 mo vs. 41.7 mo) [56]. Secondary analyses of circulating tumor cell DNA demonstrated higher rates with the conventional approach, but the clinical implications of this remain to be demonstrated. Further, chronic quality of life issues including significantly increased post-operative diarrhea with increasing resection around the SMA further question more radical approaches [57]. However, SMA-first approaches can be utilized for safe venous resection, which is often required in locally aggressive pancreatic cancer.

Venous resection and reconstruction have been advocated in both the NCCN guidelines and the American College of Surgeons’ Operative Standards, due to acceptable perioperative and oncologic outcomes [6,58,59]. All of the members of the perioperative REDISCOVER guidelines were willing to perform venous resection, demonstrating uniform acceptance [25]. However, in LAPC vein involvement is by definition not amenable to upfront resection and reconstruction, often due to occlusion. In cases with SMV/PV occlusion, collateral vein formation is common and associated with increased operative time, blood loss, and grade III+ complications (33% vs. 10% no collateral veins) [60]. Preoperative decompression with a mesenteric portal catheter or mesocaval anastomosis can be utilized. Regardless, safe venous resection and reconstruction is dictated by four core principles: (1) surrounded by air; (2) complete portal/mesenteric venous isolation; (3) maintenance of mesenteric venous drainage; (4) maintenance of left-sided drainage to the spleen and stomach [61]. In highly specialized centers with appropriate experience, venous resection may also be performed in tandem in patients with arterial involvement classified as locally advanced (~26%) [17].

One option for management of arterial involvement in LAPC is periarterial divestment, a technically demanding procedure whereby the periarterial soft tissue surrounding the vessel is completely resected preserving the integrity of the visceral arterial wall [62]. This is performed by first confirming margins on the vessel proximally and distally, then utilizing bipolar cautery to enter the periarterial dissection plane, and performing sharp dissection proximally to clear the right hemi-circumference of the SMA/circumference of the visceral artery at risk [63,64]. Periarterial divestment enables tumor resection in a select group of patients while avoiding the increased morbidity of arterial resection and reconstruction; however, one must be prepared for arterial reconstruction with every divestment. Pre-operative radiologic assessment can define the ability to perform periarterial divestment, with patients who demonstrate the so-called halo sign when the SMA is patent and remains free from invasion seen as potential candidates [63]. In comparison, the string sign (arterial contour deformity) confers arterial invasion and the need for formal resection. Periarterial divestment is associated with higher rates of post-pancreatectomy hemorrhage (17% vs. 6%) and in-hospital mortality (6% vs. 0.4%) compared to standard surgery [64]. However, these data compare favorably to arterial resection.

Arterial resection and reconstruction are increasingly being espoused as a management technique for LAPC, with half of the participants of the international perioperative guidelines willing to consider arterial resection [25]. In a series of 84 patients with LAPC who underwent surgical resection, 69.4% required arterial resection which was associated with increased 90 d mortality (8.6% vs. 2.8% venous resection vs. 2.3% no vessel resection) [65]. Arterial resection was associated with improved pathologic features and similar oncologic outcomes to patients who had no vessel resection, highlighting the highly selected nature of this population and the benefit of multimodal therapy. Furthermore, groups have shown a reduction in operative mortality with increasing experience, with an estimated learning curve of ~15 arterial resections [66]. These outcomes have been demonstrated in even the most complex cases of arterial resection when visceral interposition graft is required. In a series of 111 patients, an experienced center demonstrated a reduction in operative mortality to 5% in a contemporary period and primary patency rates of 91%, with the highest patency seen when superficial femoral artery interposition grafts were utilized (95%) [23]. These excellent outcomes have been achieved because of experienced operative surgeons, dedicated protocols for perioperative care, and avoidance of co-existing pancreaticojejunostomy [23]. However, these outcomes must be balanced with chronic quality of life issues (GI dysmotility, refractory ascites, and malabsorption) and operative mortality in a disease unlikely to be amenable to surgical cure [65]. Even more radical approaches such as intestinal auto transplantation have been described; but this approach remains investigational [67]. Arterial resection remains controversial in PDAC and was permitted in the REDISCOVER guidelines only when involvement was limited to the celiac trunk/hepatic artery and not the SMA [25].

One specific circumstance in advanced tumors that warrants further discussion are pancreatic head/body tumors with hepatic artery/celiac axis involvement. Pancreatic head tumors with isolated common hepatic artery involvement and pancreatic body/tail tumors with ≤180 degrees of celiac artery involvement are considered BRPC, while anything more advanced is considered LAPC [6]. Distal pancreatectomy with celiac axis resection is associated with an R0 resection of 91%, high morbidity (48%), but no mortality in one series [50]. The Achilles heel of this approach are gastric complications, which are the major driver of mortality. Pre-conditioning with routine coil embolization of the common hepatic artery to enlarge collaterals through the gastroduodenal arcade was shown in a case series to decrease ischemic gastropathy [50]. Classification schema based on arterial resection, need for revascularization, and type of pancreatectomy required are useful to compare outcomes and determine feasibility of gastric preservation (i.e., adequacy of prefusion via right gastric and right gastroepiploic arteries) [68]. These tumors are rare but requires technically demanding operations with meticulous pre-operative planning.

### 4.2. Radiation

The 2026 American Society for Radiation Oncology (ASTRO) guideline recommends chemoradiation or RT after multiagent chemotherapy for LAPC, with restaging before RT and resection considered afterward based on response [69]. National Comprehensive Cancer Network (NCCN) guidelines lists chemoradiation or stereotactic body radiotherapy (SBRT) after induction chemotherapy for patients without systemic metastases, reserves upfront chemoradiation or SBRT for those who are not candidates for induction chemotherapy, and accepts dose-escalated hypofractionated regimens with caution, ideally within a clinical trial [6]. The basis is local control, since no randomized trial on a modern chemotherapy backbone has shown that RT extends OS. In a rapid autopsy series, roughly a third of patients with PDAC died with locally destructive disease rather than widespread metastases [70], and local progression accounts for much of the terminal morbidity [71].

The older randomized trials predate modern systemic therapy and point in opposite directions. In FFCD/SFRO 2000-01, upfront chemoradiation (60 Gy in 30 fractions with 5-fluorouracil and cisplatin) was more toxic and less effective than gemcitabine alone (mOS: 8.6 mo vs. 13 mo) [72], whereas ECOG 4201, closed early after 74 patients, found that gemcitabine with concurrent RT (50.4 Gy in 28 fractions) improved OS over gemcitabine alone (mOS: 11.1 mo vs. 9.2 mo) [73]. SCALOP, a randomized phase II trial, supports capecitabine as the preferred concurrent agent after induction chemotherapy and identified a post-induction CA 19-9 below 46 IU/mL as a marker of benefit from chemoradiation [74,75].

LAP07 and CONKO-007 tested consolidation chemoradiation against continued chemotherapy in patients without progression on induction therapy and reached the same conclusion. In LAP07, patients controlled after 4 months of gemcitabine with or without erlotinib were randomized to chemoradiation (54 Gy in 30 fractions with capecitabine) or continued chemotherapy [30]. OS did not differ (mOS: 15.2 mo vs. 16.5 mo, *p* = 0.83), but chemoradiation reduced local progression (32% vs. 46%, *p* = 0.03). Its radiation quality assurance, sometimes questioned, was prospective and centrally reviewed; major deviations occurred in 18% of plans, too few to explain the result [76]. The substantive limitations are a non-escalated dose (biologically effective dose (BED10) 63.7 Gy) and a gemcitabine backbone [69]. In CONKO-007, 336 patients without progression after 3 months of induction chemotherapy (predominantly FOLFIRINOX) were randomized to chemoradiation (50.4 Gy in 28 fractions with gemcitabine) or continued chemotherapy [27]. Slow accrual forced a change in primary endpoint from OS to overall R0 resection rate; neither differed (R0 25% vs. 18%, *p* = 0.11; mOS: 15 mo vs. 14 mo, HR 0.94), but among resected patients, chemoradiation increased the R0 rate (69% vs. 50%, *p* = 0.04) and reduced first-site locoregional recurrence (30% vs. 41%) [27,69]. Taken together, conventionally dosed chemoradiation improves local control and margin status without extending survival; whether an ablative dose changes that is the open question [69,77].

The 50–54 Gy used in these trials (BED10 roughly 60–64 Gy) reflects what the adjacent duodenum, stomach, and bowel tolerate, not what an adenocarcinoma requires. An ablative dose (BED10 near 100 Gy) can be reached with 67.5 Gy in 15, 75 Gy in 25, or 50 Gy in 5 fractions; the common SBRT schedules of 33–40 Gy in 5 fractions are not ablative despite the label [77]. The evidence is mostly retrospective. In the first series to raise the question, 47 of 200 patients with tumors more than 1 cm from luminal organs received a BED10 above 70 Gy and lived longer than those treated conventionally (3-year OS 31% vs. 9%) without added toxicity [78]. A 119-patient cohort treated to a BED10 near 98 Gy on standard linear accelerators reported median OS of 18.4 months from RT, 2-year locoregional failure of 33%, and grade III gastrointestinal bleeding in 8% [79].

The prospective evidence is single-arm. The multi-institutional phase II SMART trial treated 136 patients with LAPC or BRPC to 50 Gy in five fractions with MR-guided online adaptive RT after induction chemotherapy, met its primary endpoint of no acute grade III+ gastrointestinal toxicity definitely attributed to treatment, and reported 2-year local control and OS from RT of 78% and 41% [80,81]. MAIBE, a single-institution phase II trial, treated 48 patients still unresectable after induction chemotherapy to 67.5 Gy in 15 or 75 Gy in 25 fractions with capecitabine; 2-year OS from RT was 38% overall and 54% in the 27% who were subsequently resected [82]. These figures exceed the 2-year OS of under 25% in CONKO-007 [27,77], but the patients were selected, and single-arm data cannot say by how much. A retrospective comparison of 211 unresected patients found lower 2-year local failure with 5-fraction MR-guided adaptive RT than with 15/25-fraction CT-guided RT (6.5% vs. 33%) but no significant OS difference [83]. In a disease dominated by distant failure, local control has not yet been shown to translate into improved survival.

An ablative dose adjacent to the duodenum is safe only when the position of the target and luminal organs is known at each fraction and the plan can respond to it. ASTRO lists dose-escalated regimens as an option but requires motion management and daily volumetric image guidance, recommends online adaptive replanning for dose-escalated SBRT, and accepts moderate hypofractionation with selective offline replanning where online adaptation is unavailable [69]. A 25-fraction course tolerates a tumor abutting bowel better than 5 fractions without daily adaptation, whereas MR guidance permits tighter margins and on-table replanning [69]. Luminal organ constraints take priority over target coverage, and gross tumor invasion of the stomach or duodenum warrants caution; NCCN advises against SBRT in that setting [6,69]. Elective coverage of the peritumoral region, perineural tracts, and regional nodes is recommended at a non-escalated dose [69,84]. Dose-escalated RT for LAPC should ideally be delivered at centers with an established program, volumetric image guidance, motion management, and the ability to adapt treatment when anatomy requires it [6,69,77]. Where these are unavailable, conventionally fractionated chemoradiation and non-escalated SBRT remain appropriate, guideline-endorsed options [6,69].

Selection for ablative RT mirrors surgical selection. Ideal candidates have completed several months of multiagent chemotherapy with stable disease on imaging and a decline in CA 19-9, have no distant progression, and have tumors that do not invade luminal organs [69,77]. A rising CA 19-9 without a radiographic explanation should prompt additional investigation, potentially including diagnostic laparoscopy, before committing to local therapy [77]. Surgery after ablative RT carries specific risks. In SMART, 35% of patients underwent surgery, about half with vascular resection; grade III+ complications occurred in 20.5%, and two postoperative deaths were possibly related to RT, prompting caution with vascular resection after ablative SBRT [80,81]. In MAIBE, 13 of 48 patients (27%) were resected with no 90-day mortality [82]. A 34-patient series of exploration after ablative RT reported aborted resection in 26%, vascular resection or divestment in 56%, 90-day mortality of 5.9%, and clinically significant ascites in 36% [85]. Ascites after resection appears in both reports. ASTRO recommends surgery 4–8 weeks after RT and surgical input before dose escalation in any patient who may proceed to resection [69].

Randomized trials of dose escalation are underway. NRG-GI011 (LAP100) randomizes patients with LAPC who have completed 4–8 months of chemotherapy without progression and with a CA 19-9 response to dose-escalated RT (50 Gy in 5 or 75 Gy in 25 fractions) or standard care, with 3-year OS as the primary endpoint [86]. The Dutch LAPSTAR trial randomizes patients after at least 2 months of chemotherapy to MR-guided 50 Gy in five fractions or standard care, with quality-of-life deterioration-free survival as the primary endpoint [87]. However, the case for RT in LAPC is a local control benefit and with a suggestion of a survival signal with ablative dosing based on retrospective and single-arm data, both contingent on patient selection and safe delivery [69,77]. However, there is no Level 1 data to date that consistently demonstrate an overall survival benefit to ablative radiation.

### 4.3. Additional Locoregional Modalities

Beyond surgical resection and radiation, additional therapies such as thermoablation, tumor-treating fields (TTF), intra-arterial chemotherapy, and irreversible electroporation (IRE) have all been or are currently under study in LAPC. The PELICAN trial was a randomized phase III trial that evaluated patients with unresectable LAPC that had received induction chemotherapy to radiofrequency ablation or chemotherapy alone [88]. The primary outcome of the trial, overall survival, was similar between arms (mOS: 12.1 mo vs. 11.6 mo). Furthermore, PFS was also similar and there were more grade III+ adverse events and decreased quality of life in the RFA arm, suggesting RFA does not have a role in LAPC. On the other hand, PANOVA-3 was also a phase III trial in upfront LAPC which randomized patients to GA +/− TTF (device delivered ≥18 h/day) [89]. OS, the primary outcome, was increased (mOS: 16.2 mo vs. 14.2 mo, HR: 0.82, 95% CI: 0.68–0.99, *p* = 0.039), with no difference in PFS and ORR. Safety signals demonstrated skin reaction in more than three-quarters of patients; however, most were mild with 7.7% suffering grade III+ toxicity. TTF earned FDA approval in 2026 for the first line treatment of LAPC in combination with GA based on these data. Another promising therapy is targeted intra-arterial gemcitabine, which is currently under study in the phase III TIGeR-PaC trial. Interim analysis demonstrated improved safety (adverse events: 85 vs. 245) and tolerability compared to IV gemcitabine-abraxane, with suggestion of potentially improved OS (16 mo vs. 10 mo) [90]. Irreversible electroporation is another modality that has been shown in non-randomized series be effective in LAPC and holds theoretical benefits over thermoablation [91]. Initial results from the ongoing DIRECT study comparing IRE to chemotherapy alone demonstrated similar safety data, while efficacy and quality of life data are still pending [92]. However, there are remaining safety concerns about IRE, regarding increased pancreatic fistula and mortality related to bleeding. Finally, histotripsy, high-intensity focused ultrasound waves to treat tumors, is being explored prospectively in locally advanced or oligometastatic pancreatic cancer [93]. This promising therapy could potentially revolutionize locoregional management of pancreatic cancer. Novel locoregional strategies in LAPC are required to further improve outcomes.

## 5. Future Directions

Improvements in standardization of perioperative care, assessment of disease response, novel systemic agents, and improved selection are all required to optimize outcomes in LAPC. Operative and perioperative standards in surgery allows for accurate efficacy assessment within complex pancreatic surgery, especially with respect to vascular pancreatic surgery [58]. However, standardization is critical in all phases of multimodal therapy, including pathology, radiology, radiation oncology, and medical oncology, among others [3,20,94]. Novel tools to assess treatment response and better define micro-metastatic disease are in the pipeline, with prospective studies required to confirm the predictive nature of these liquid biopsy techniques [95]. Techniques such as ctDNA and microRNA offer the potential to monitor response, detect therapeutic resistance, and even provide for early diagnosis [95,96]. In addition to the revolutionary KRAS-targeted therapies discussed previously, early studies have shown efficacy of personalized RNA vaccines in PDAC [97]. Another pancreatic cancer vaccine concept studied a GM-CSF secreting allogenic pancreatic cancer vaccine in a phase II trial in combination with immunotherapy and SBRT in LAPC, demonstrating promising efficacy data including an overall resection rate of 44% and mOS reaching beyond 3 years among resected patients [98]. These advances offer potential to revolutionize the care of patients with LAPC as they are translated from the metastatic setting.

## 6. Conclusions

Locally advanced pancreatic cancer is a challenging disease to care for due to the complex tumor–vessel involvement requiring complex surgery when indicated, all the while understanding the high-risk of systemic failure. Appropriately staging patients to define not only anatomical but also biologic aggressiveness in the individual patient who presents to clinic is paramount to optimal management. Upfront multi-agent systemic therapy regimens are recommended for all patients with LAPC; however, the era of targeted therapy in PDAC offers the potential for improved treatment paradigms. Currently, only a small minority of patients with LAPC after upfront systemic therapy ever become surgical candidates, but that number is likely to increase with effective targeted therapy. Balancing the radical surgical approach required to obtain margin negative resection with increased perioperative morbidity and mortality is required. Additional locoregional therapies should be included in a multimodal approach to select the correct treatment for the ideal patient. Critical to every decision in the management of LAPC is an effective multi-disciplinary team.

## Figures and Tables

**Table 1:** NCCN Classification of Borderline Resectable and Locally Advanced Pancreatic Cancer.

	Borderline Resectable	Locally Advanced
**NCCN**		
**Arterial**	*Pancreatic head/uncinate process:* - Solid tumor contact with CHA without extension to CA or hepatic artery bifurcation allowing for safe and complete resection and reconstruction. - Solid tumor contact with the SMA ≤ 180° - Solid tumor contact with variant arterial anatomy (eg., accessory right hepatic artery, replaced right hepatic artery, replaced CHA, and the origin of replaced or accessory artery) and the presence and degree of tumor contact should be noted if present, as it may affect surgical planning *Pancreatic body/tail:* - Solid tumor contact with the CA of ≤180°	*Pancreatic head/uncinate process:* - Solid tumor contact >180° with SMA or CA *Pancreatic body/tail:* - Solid tumor contact of >180° with the SMA or CA - Solid tumor contact with CA and aortic involvement
**Venous**	- Solid tumor contact with the SMV or PV of >180°, contact of ≤180° with contour irregularity of the vein but suitable vessel proximal and distal to the site of involvement allowing for safe and complete resection and vein reconstruction - Solid tumor contact with the inferior vena cava (IVC)	- Not currently amenable to resection and primary reconstruction due to complete occlusion of SMV/PV

Common hepatic artery (CHA); celiac axis (CA); superior mesenteric artery (SMA); superior mesenteric vein (SMV); portal vein (PV); inferior vena cava (IVC). NCCN Version 4.2026 [6].

## Data Availability

All data used in publication is published and publicly available.

## Abbreviations

Pancreatic ductal adenocarcinoma (PDAC); locally advanced pancreatic cancer (LAPC); borderline resectable pancreatic cancer (BRPC); Duke pancreatic monoclonal antigen type 2 (DUPAN-2); Gemcitabine/albumin-bound paclitaxel (GA); modified leucovorin/fluorouracil/irinotecan/oxaliplatin (mFOLFIRINOX/FOLFIRINOX); liposomal irinotecan/fluorouracil/leucovorin/oxaliplatin (NALIRIFOX); median overall survival (mOS); objective response rate (ORR); superior mesenteric artery (SMA); superior mesenteric vein (SMV); portal vein (PV); radiation therapy (RT); American Society of Radiation Oncology (ASTRO); National Comprehensive Cancer Network (NCCN); stereotactic body radiotherapy (SBRT); biologically effective dose (BED10); tumor-treating fields (TTF); irreversible electroporation (IRE).
